# From Sedation to Injury: Isoflurane‐Associated Hepatic Dysfunction: A Series of 3 Intriguing Cases

**DOI:** 10.1002/ccr3.73838

**Published:** 2026-11-02

**Authors:** Mohammad Nabeel Aamir Syed, Faina Qaiser, Fabeha Hilal Makhdoomi, Muhammad Taha Arshad Shaikh, Syeda Aisha, Tanvir Hayder

**Affiliations:** ^1^ A. O. Hospital Karachi Pakistan; ^2^ Abbasi Shaheed Hospital Karachi Pakistan; ^3^ Sindh Government Children Hospital Karachi Pakistan; ^4^ Department of Plastic Surgery Shaheed Mohtarma Benazir Bhutto Institute of Trauma Karachi Pakistan; ^5^ University of Dhaka Dhaka Bangladesh

**Keywords:** case series, hepatitis, hepatotoxicity, isoflurane, transaminitis

## Abstract

Hepatitis occurring post‐induction of general anesthesia remains a perplexing conundrum of medicine. Isoflurane, one of the most commonly employed drugs in the maintenance of anesthesia worldwide, has succeeded outdated agents such as halothane. Despite its widespread and high frequency of usage, there exist unanticipated and undesirable side effects and toxicity that are usually overlooked, as the symptoms are mostly generic and insidious. It is reported that isoflurane causes acute liver injury ranging from mild transaminitis to fulminant hepatic failure. This case series discusses patients who have been unfortunate victims of this situation, developing severe transaminitis after exposure to isoflurane during general anesthesia induction and maintenance for surgical interventions for their respective ailments in the absence of any other relevant causal links. Every patient presented with postoperative jaundice and markedly elevated liver enzymes, which were treated conservatively, resulting in full recovery. In the absence of more specific therapies, conservative management remains the cornerstone of treatment. In this article, 3 intriguing cases have been described. These cases highlight the need for more research and greater awareness of isoflurane‐induced hepatotoxicity, especially given its non‐specific presentation.

## Key Clinical Message

Isoflurane; a widely used agent for anesthetic induction and maintenance. Rare postoperative hepatic dysfunction secondary to exposure is a rare but valid occurrence that may manifest as worsening liver function postoperatively, urging clinicians to remain vigilant regarding this potentially fatal side effect and plan early intervention to prevent poor outcomes.

## 1 Introduction

Isoflurane is a volatile anesthetic used during the induction and maintenance of general anesthesia. Like most volatile anesthetics, isoflurane is a halogenated ether compound. It is a structural isomer of enflurane, favored for its low cardiotoxicity, minimal systemic side effect profile, and minimal hepatic metabolism. Unlike older agents like halothane, only about 2% of isoflurane undergoes hepatic metabolism, making it a common and preferred choice in various surgical settings [1].

Despite its favorable safety profile, there have been reports of isoflurane‐associated liver injury. The incidence of isoflurane‐associated hepatotoxicity is extremely rare, estimated to be < 1 in 1,000,000 patients, far less than traditional anesthetic agents [2]. Severity ranges from mild, transient, and asymptomatic transaminitis to severe, acute hepatic failure [3, 4].

The exact mechanism of isoflurane‐induced hepatotoxicity is unclear; however, it is believed to work similarly to halothane toxicity and involves the production of reactive by‐products like trifluoroacetyl (TFA) intermediates, which may trigger an immune response [4].

Due to its rarity and the nonspecific nature of symptoms, isoflurane‐induced liver injury is often misdiagnosed or overlooked. Risk factors may include metabolic conditions like diabetes, previous exposure to halogenated anesthetics, or altered immune regulation [5, 6].

In this case series, we present three patients who developed severe transaminitis following exposure to isoflurane during general anesthesia, without any other identifiable cause. All were managed conservatively with supportive care and achieved full clinical recovery. Through these cases, we aim to highlight the importance of recognizing isoflurane as a potential, though rare, cause of acute liver injury in the postoperative setting.

## 2 Case Presentations

### 2.1 Case No.1

A male in his mid‐50s, with a known case of diabetes mellitus and no known drug allergies, presented to the emergency department with lower abdominal pain, vomiting, and absolute constipation for 2 days. On physical examination, the abdomen was noted to be diffusely tense and tender. Chest and erect abdominal radiographs revealed pneumoperitoneum, suggestive of a perforated viscus. The patient was vitally stable and was prepared for emergency laparotomy following baseline investigations, including Complete Blood Count (CBC), Prothrombin time/International Normalized Ratio (PT/INR), Liver Function Tests (LFTs), Urea, Creatinine and Electrolytes (UCEs), and viral markers. The results were unremarkable except for mildly elevated Total Leukocyte Count (TLC) with 90% neutrophils. At baseline, the patient had no derangements in bilirubin levels or INR, no underlying chronic liver disease, no history of alcohol misuse, and no signs of malnutrition, congestive heart failure, shock, or any other underlying hematologic and hepatobiliary disorder, successfully ruling out any potential causes of liver insult preoperatively.

General anesthesia was induced with intravenous Propofol and maintained with Isoflurane and Atracurium besylate. Laparotomy revealed a perforated duodenum secondary to a duodenal ulcer. The intraoperative course was unremarkable, and the patient was managed in the ICU postoperatively. No blood product transfusions were required, and no significant blood loss was reported intraoperatively. Additionally, the patient sustained no injury to his liver and developed no infection of the tissue spaces or viscera.

Inhalational isoflurane was administered at an initial concentration of 0.5%, and gradually increased to 2% during induction; maintenance was provided with a 3% concentration throughout the procedure for 2 h. Intravenous propofol was administered during anesthesia induction at a dose of 1 mg/kg and atracurium was administered via a loading dose of 0.5 mg/kg and was titrated at 1 mg/kg/h. The analgesics used were intravenous paracetamol 1 g TDS and intravenous tramadol 50 mg BD; the antibiotic given was intravenous meropenem 1 g BD. No specific ICU medications were given. The medications administered were all according to the concentrations approved by regulatory authorities and literature, thus making hepatotoxicity less likely.

On the second postoperative day, the patient developed scleral icterus and a dramatic rise in liver enzymes, Alanine Aminotransferase (ALT = 5530 U/L), Alkaline Phosphatase (ALP = 302 U/L), direct bilirubin (3.2 mg/dL), and PT (16 s) and INR (2.2), suggestive of acute liver injury. Abdominal ultrasonography revealed altered hepatic parenchyma and a contracted gallbladder.

The patient was managed conservatively with close monitoring. By the 17th postoperative day, his ALT dropped to 292 U/L. Bilirubin and PT/INR also showed progressive improvement. By the 25th postoperative day, the patient had achieved full clinical recovery.

### 2.2 Case No.2

A male in his 30s, with no known comorbidities, presented to the emergency department with a one‐day history of diffuse abdominal pain and absolute constipation. On clinical examination, the abdomen was noted to be tense and distended. Bowel sounds were absent on auscultation. Abdominal radiography revealed multiple air‐fluid levels, suggestive of an intestinal obstruction. The past surgical history was remarkable for an open appendectomy due to an acute appendiceal perforation 10 years ago.

The patient was vitally stable and was prepared for emergency laparotomy following baseline investigations, including CBC, PT/INR, UCE, LFTs, and viral markers. The results were unremarkable. Yet again, at baseline, the patient had no derangements in bilirubin levels or INR, no underlying chronic liver disease, no history of alcohol misuse, and no signs of malnutrition, congestive heart failure, shock, or any other underlying hematologic and hepatobiliary disorder, successfully ruling out any potential causes of liver insult preoperatively.

General Anesthesia was induced with intravenous Propofol and maintained with Isoflurane, Oxygen, and Atracurium besylate.

Intraoperatively, multiple intestinal adhesions were found and released, and resection and anastomosis of the affected segment were performed. A surgical drain was placed in the peritoneal cavity for postoperative drainage. The intraoperative course was uneventful, and the patient was managed in the ICU postoperatively. No blood product transfusion was required, and no significant blood loss was reported; additionally, the patient sustained no intraoperative liver injury.

Inhalational isoflurane was administered at an initial concentration of 0.5%, and gradually increased to 2% during induction; maintenance was provided with a 3% concentration throughout the procedure for 1.5 h. Intravenous propofol was administered during anesthesia induction at a dose of 1 mg/kg and atracurium was administered via a loading dose of 0.5 mg/kg and was titrated at 1 mg/kg/h. The analgesics used were intravenous paracetamol 1 g TDS and intravenous tramadol 50 mg BD; the antibiotic given was intravenous meropenem 1 g BD. No specific ICU medications were given. The medications administered were all according to the concentrations approved by regulatory authorities and literature, thus making hepatotoxicity less likely.

On the 3rd postoperative day, the patient developed severe abdominal pain with generalized tenderness. There was evidence of fecal contamination of the peritoneal cavity, as evidenced by the drain contents containing fecal matter. The patient was again prepared for emergency laparotomy, yet again induced with intravenous Propofol and maintained with Isoflurane, Oxygen, and Atracurium besylate.

Emergency exploratory laparotomy revealed an anastomotic duodenal perforation. The procedure proceeded uneventfully. This time, an ileostomy was created. Postoperatively, the patient was managed in the ICU and was subsequently shifted to the ward. The patient displayed no signs of infection or shock and otherwise appeared clinically well.

On the subsequent 3rd postoperative day, the patient developed scleral icterus. Laboratory investigations showed a dramatic spike in liver enzymes: ALT (3778 U/L), elevated bilirubin (direct bilirubin 7.4 mg/dL), ALP (1777 U/L), and PT (18 s) and INR (1.57), raising concerns for acute liver injury.

With strong supportive care, the patient's liver function improved. By the 16th postoperative day, ALT dropped to 170 U/L, and bilirubin and PT/INR also showed progressive normalization, and the patient achieved complete clinical recovery by the 20th postoperative day.

### 2.3 Case No.3

A male in his late 60s, with no relevant underlying health issues or surgical history, presented to the emergency department for assessment of severe epigastric pain and vomiting, which had occurred over the previous 2 days. Notably, he was taking no NSAIDs, alcohol, or hepatotoxic medicines. The only recurrent treatment was homeopathic remedies for epigastric pain containing *Iris versicolor* and Magnesium Phosphate, neither of which contributes to liver insult.

On examination, the patient was alert but appeared uncomfortable. Upon assessment, vital signs at presentation were within normal limits. Examination of his abdomen revealed diffuse tenderness, guarding, and absent bowel sounds. A chest radiograph revealed free air underneath the diaphragm, consistent with perforation of a hollow viscus. Laboratory workup demonstrated a moderately elevated white blood cell count (WBC 17 × 10^9^/L) with 92% neutrophils. Furthermore, at baseline, the patient had no derangements in bilirubin levels or INR, no underlying chronic liver disease, no history of alcohol misuse, and no signs of malnutrition, congestive heart failure, shock, or any other underlying hematologic and hepatobiliary disorder, successfully ruling out any potential causes of liver insult preoperatively.

An emergency exploratory laparotomy was performed. Induction of general anesthesia was performed with intravenous Propofol and Rocuronium, and maintained with Isoflurane. During the procedure, a minor perforation was identified on the anterior wall of the first part of the duodenum and repaired. No evidence of active bleeding was seen. Other abdominal viscera were grossly normal. The intraoperative course was unremarkable, and subsequently, the patient was transferred to the ICU for postoperative monitoring.

Inhalational isoflurane was administered at an initial concentration of 0.5%, and gradually increased to 2% during induction; maintenance was provided with a 3% concentration throughout the procedure of 2.5 h. Intravenous propofol was administered during anesthesia induction at a dose of 1 mg/kg, and rocuronium was administered via a loading dose of 0.6 mg/kg and was titrated at 7 μg/kg/min. The analgesics used were intravenous paracetamol 1 g TDS and intravenous tramadol 50 mg BD; the antibiotic given was intravenous meropenem 1 g BD. No specific ICU medications were given. The medications administered were all according to the concentrations approved by regulatory authorities and literature, thus making hepatotoxicity less likely.

On the 2nd postoperative day, the patient developed scleral icterus. Liver function tests were significantly deranged, with a sharp rise in ALT (2160 U/L), ALP (231 U/L), total bilirubin (1.9 mg/dL with 1.4 direct bilirubin), and INR (1.46). The findings were consistent with acute hepatocellular injury. Additional labs revealed elevated serum urea (103 mg/dL) and elevated potassium (6.2 mEq/L), which returned to normal with supportive treatment.

One unit of fresh frozen plasma was transfused and conservatively managed with close lab monitoring. Serial LFTs showed progressive improvement, and by the 5th postoperative day, the patient was clinically stable and transferred to an HDU. The liver enzymes continued on a downward trend over the next few weeks and returned to near‐normal values.

## 3 Differential Diagnoses

The differential diagnosis of acute postoperative liver injury included ischemic hepatitis, acute viral hepatitis, biliary obstruction, sepsis‐associated hepatic dysfunction, autoimmune hepatitis, and drug‐induced liver injury secondary to other perioperative hepatotoxic medications. Ischemic hepatitis was considered unlikely, as none of the patients had documented perioperative hypotension, shock, significant blood loss, congestive cardiac failure, or other evidence of hepatic hypoperfusion. Acute viral hepatitis was investigated with preoperative viral markers, which were unremarkable, while the absence of clinical or radiological evidence of hepatobiliary obstruction argued against an obstructive etiology. Sepsis‐related hepatic dysfunction was also considered but was not supported by the clinical course or absence of postoperative shock or significant systemic infection. We did not identify other recognized hepatotoxic exposures in the available histories. Inquiries regarding any familial causes of hepatotoxicity or liver failure proved to be unremarkable. Although postoperatively raised intra‐abdominal pressure may result in deranged liver enzymes, serial abdominal examinations performed revealed no signs of the said condition, making it an unlikely cause. Across all three cases, liver function tests were normal before anesthesia, marked hepatocellular injury developed within 2–3 days of isoflurane exposure, and subsequent biochemical improvement occurred following supportive management. The close temporal association, exclusion of alternative causes, and favorable course following cessation of exposure collectively support isoflurane‐associated drug‐induced liver injury as the most plausible diagnosis, although definitive causality cannot be established in a case series and formal causality assessment using scores was not performed.

The underlying abdominal conditions also warrant consideration when interpreting these cases: Case 1 involved a perforated duodenal ulcer; Case 2 involved intestinal obstruction requiring resection and anastomosis followed by an anastomotic perforation with fecal contamination, and Case 3 involved a perforated duodenal lesion. Although the recorded clinical course did not document shock, these acute surgical illnesses and the possibility of evolving infection or inflammatory stress are relevant competing explanations. The ICU setting makes concomitant drug exposure plausible, including the administration of possible hepatotoxic agents. Even in the ICU setting, the medications given were controlled. Analgesics included paracetamol and tramadol given well within safe doses. The antibiotic given was meropenem to all three patients, which carries minimal risk of hepatotoxicity. The anesthetic agents used were atracurium and propofol in Cases 1 and 2, and rocuronium and propofol in Case 3; given within safe doses.

## 4 Discussion

Isoflurane, a halogenated ether, received FDA approval in 1979 for use in both the induction and maintenance of general anesthesia. It is a structural isomer of enflurane, nonflammable in nature, but its utility for inhalational induction of anesthesia is limited due to its pungent odor. The anesthetic action occurs through modulation of GABA, glycine, and NMDA receptors, resulting in sedation, amnesia, and immobility [7].

Isoflurane is a widely employed agent for the maintenance of general anesthesia owing to its minimal side effect profile and potent anesthetic effects. Nevertheless, the principles of pharmacodynamics do not render any drug entirely free from adverse effects. Similar is the case with isoflurane. It has been observed that exposure to the inhalational anesthetic subsequently leads to an alteration of the liver enzymes immediately postoperatively, followed by a return to baseline within 24 h [8]. Although rare, isoflurane‐associated hepatotoxicity manifests as an acute liver injury longer than the expected benign 24‐h course in the postoperative period following induction and maintenance of general anesthesia [5, 8]. Consequently, caution is advised in the patient population already afflicted with any form of insult to the liver.

The liver is well‐established as the primary organ for drug metabolism and detoxification. The primary goal of drug metabolism is to produce water‐soluble intermediates that can be readily eliminated by the body's renal system, thereby preventing drug accumulation to toxic doses and, consequently, preventing any adverse effects. It becomes concerning when these metabolites themselves carry either a toxic or immunogenic nature, causing widespread liver inflammation. Such an incidence is relatively rare among patients exposed to isoflurane (0.2%) when compared with other inhalational anesthetic agents; nevertheless, its existence and potential to cause harm cannot be overlooked [9].

The most frequent insult to the liver is usually moderate to severe transaminitis; however, cases of fulminant hepatic failure following isoflurane exposure have also been documented [10]. Hepatic injury following isoflurane exposure may occur even in the absence of a history of prior exposure to halogenated inhalational agents. Nevertheless, there is stronger evidence to suggest a positive correlation with prior exposure or underlying immune dysregulation [5].

Milder examples include a report by Malnick et al. [11] where a patient developed acute cholestatic hepatitis 3 weeks after exposure to isoflurane. The patient initially had markedly abnormal liver enzymes and bilirubin levels, but improved gradually over several months.

A more severe case by Peiris et al. [10] included a fatal report, in which a 77‐year‐old patient developed liver failure within 2 days of Isoflurane anesthesia for hemicolectomy, despite no confirmed history of prior sensitization.

Turner et al. [5] described a 72‐year‐old woman who developed fulminant hepatic necrosis 6 days after knee amputation under isoflurane. She had been exposed to isoflurane 3 years earlier, raising the possibility of a sensitization reaction and an immune‐mediated mechanism of liver injury.

Brunt et al. [12] reported a 39‐year‐old woman who developed fulminant hepatic failure after repeated exposures to isoflurane, requiring a liver transplant. Histology revealed massive hepatic necrosis resembling the histological picture of halothane hepatitis.

Therefore, it might very well be established that a spectrum of liver injuries and a plethora of clinical presentations and outcomes exist following exposure to isoflurane. Additionally, it proves to be challenging to predict the precise pattern a certain level of exposure may take or if a history of prior exposure could form a stronger causal link.

Since an antidote is still nonexistent, along with any specific treatment regimens, conservative management remains the cornerstone of therapy. There have been a few notable attempts at studying the root cause and addressing it at the molecular level, in addition to supplementation with vitamin B‐1. However, these advances are still in the experimental phase, undergoing animal trials, and further extensive studies need to be conducted before a specific treatment is discovered [13, 14].

In this case series, we describe three patients who developed severe transaminitis shortly after exposure to isoflurane during general anesthesia, in the absence of other identifiable causes. Despite the varying degrees of liver injury, from moderate to severe, all patients responded well to conservative management and achieved complete recovery. These findings reinforce that although isoflurane is generally regarded as a safe volatile anesthetic with minimal hepatic metabolism, it can rarely be associated with significant liver injury. Furthermore, existing evidence from previous reports establishes that isoflurane‐related liver injury can vary widely, from mild self‐resolving hepatitis to severe life‐threatening liver failure.

Even though recent studies have not reported a rising trend in the incidence of isoflurane‐associated hepatitis, the condition still warrants a pragmatic approach and vigilant weighing of the patient's risk‐to‐benefit ratio concerning the optimal choice of inhalational anesthetic agent. Alarming reports of rising incidence have also been issued, raising valid concerns over this matter [13]. This discrepancy among clinical outcomes following exposure to isoflurane warrants further extensive study and risk stratification of the affected population, alongside clinical safety trials and prospective studies to elaborate and solve the mystery behind this complication, allowing clinicians on a global scale to form more informed decisions regarding anesthetic choice in light of patient non‐maleficence.

## 5 Conclusion

Isoflurane, a novel and reliable agent for general anesthesia maintenance, holds merit among the other available drugs. Nevertheless, its unique traits do not absolve it from producing adverse effects. Hepatotoxicity and subsequent hepatic dysfunction, however rare, ensue post‐exposure and might progress towards fulminant hepatic failure if not caught in time. Diagnosis and therapy prove challenging for the medical fraternity due to the obscure nature of the affliction and the lack of a proper, specific regimen. Conservative treatment remains the cornerstone of therapy. Further extensive research is required to conduct an in‐depth study of this rare complication and subsequently develop targeted treatment regimens.

## 6 Limitations

This case series has several limitations. The small number of cases and the retrospective nature of the observations limit the generalizability of the findings and preclude definitive conclusions about the incidence of risk factors for isoflurane‐associated hepatotoxicity. Although major alternative causes of acute liver injury were clinically and biochemically assessed, a formal causality assessment such as the RUCAM score was not performed, and the contribution of individual perioperative medications cannot be excluded with absolute certainty. Prior anesthetic exposure and detailed past surgical history need to be consistently documented across all cases to assess possible sensitization after previous exposure. Furthermore, liver biopsy and comprehensive immunological investigations were not performed, limiting pathological confirmation of the diagnosis. Finally, the absence of a control group prevents direct comparison with patients undergoing similar procedures without isoflurane exposure. These limitations support interpreting the cases as temporally associated with isoflurane exposure rather than proof of causation, and highlight the need for larger prospective studies with complete medication records, documented anesthetic exposure parameters, standardized causality assessment, and appropriate comparator groups to better establish the relationship between isoflurane exposure and acute liver injury.

## Author Contributions

**Mohammad Nabeel Aamir Syed:** conceptualization, supervision. **Faina Qaiser:** writing – original draft. **Fabeha Hilal Makhdoomi:** writing – original draft. **Muhammad Taha Arshad Shaikh:** project administration. **Syeda Aisha:** writing – review and editing. **Tanvir Hayder:** writing – review and editing.

## Funding

The authors have nothing to report.

## Ethics Statement

The authors have nothing to report.

## Consent

Appropriate verbal and written informed consent was obtained from the patient presented in the report. Written informed consent from all the patients was obtained according to journal guidelines.

## Conflicts of Interest

The authors declare no conflicts of interest.

## Data Availability

Data sharing not applicable to this article as no datasets were generated or analysed during the current study.
